# Effects of acute and long-term mindfulness on neural activity and the conflict resolution component of attention

**DOI:** 10.3389/fnhum.2024.1359198

**Published:** 2024-02-21

**Authors:** Dehan Elcin, Miguel Velasquez, Paul J. Colombo

**Affiliations:** ^1^Department of Psychology, Tulane University, New Orleans, LA, United States; ^2^Tulane Brain Institute, Tulane University, New Orleans, LA, United States

**Keywords:** mindfulness, conflict resolution, Stroop, fNIRS, meditation

## Abstract

Mindfulness practices have been linked to enhanced attention and conflict resolution abilities. While much research has focused on the long-term effects of mindfulness, the immediate impact of a single session has been less studied. This study recruited 20 experienced meditators and 20 novices and assigned them to a mindfulness or a control condition. They completed a Stroop Task to measure cognitive conflict resolution before and after the intervention, with brain activity monitored via functional near-infrared spectroscopy (fNIRS). Novices showed an age-related decline in conflict resolution ability, while experienced meditators didn’t. Initially, both groups showed similar Stroop performance, but experienced meditators had greater brain activation in the left dorsolateral prefrontal cortex (DLPFC). Post-intervention, novices in the breath count task became more similar to experienced meditators in their neural activity during conflict resolution. Our findings indicate that long-term mindfulness experience may protect against age-related decline in cognitive conflict resolution speed, and may alter neural processing of cognitive conflict resolution.

## 1 Introduction

Conflict resolution is a crucial part of the executive component of the attention system as proposed by Posner and Petersen (Posner and Petersen, 1990; Petersen and Posner, 2012). This skill includes managing and resolving conflicting information or competing cognitive processes and its successful execution is integral to everyday life activities such as decision-making, problem-solving, and interpersonal communication (Zaki et al., 2010). Conflict resolution performance is often investigated by measuring the speed and accuracy of processing conflicting sensory information on tests such as Stroop (1935) and Simon (1969), flanker (Eriksen and Eriksen, 1974) and the attention network task (Fan et al., 2002).

Mindfulness practices have grown in popularity and have been reported to improve attention skills, particularly in the sphere of cognitive conflict resolution. Multiple studies have shown that experienced meditators, with a wide range of experience (82–14,000 days), process incongruent trials of the Stroop task, which involve sensory conflict resolution, faster than novices (Chan and Woollacott, 2007; Moore and Malinowski, 2009; Fabio and Towey, 2018). Meditators with a wide range of experience (4–360 months) have also demonstrated increased processing speed on the executive component attention network task, which also specifically measures speed of conflict resolution (Jha et al., 2007; Jo et al., 2016). Previous studies have also demonstrated improvements in novices after brief periods of mindfulness training. Novices show increased processing speed on the Stroop task after 5 days of mindfulness training, (Fan et al., 2014), and both increased processing speed and decreased N2-event-related amplitude on a modified Stroop task, after 8-weeks of mindfulness training (Malinowski et al., 2015). There have been few studies focusing on single-interventions on beginners, all showing increased processing speed of incongruent trials after a single-mindfulness intervention (Wenk-Sormaz, 2005; Deepeshwar et al., 2015; Izzetoglu et al., 2020; Sleimen-Malkoun et al., 2023). Only one of these studies has examined the effect of a single mindfulness intervention on the conflict resolution ability of experienced meditators and novices (Sleimen-Malkoun et al., 2023). That study demonstrated that meditators with 4 + months of regular meditation practice and novices who completed a 10 min mindfulness session processed both incongruent and congruent trials of a Stroop task faster than those who completed an active listening task indicating both an improvement in cognitive conflict resolution, and processing speed.

Previous studies report an age-related decline in speed of cognitive conflict resolution (Davidson et al., 2003). Long-term mindfulness practice has been linked with a slowing of this decline in processing speed. One study found older Vihangam Yoga meditation practitioners to show decreased Stroop interference compared to an age-matched control group (Prakash et al., 2012). Another interventional study reported a reduction in Stroop interference in an older population after a 7-week mindfulness meditation intervention (Oken et al., 2010). There are other studies that report null-findings (Wetherell et al., 2017), however, and the efficacy of mindfulness training in slowing cognitive decline remains a question.

Prior research has implicated several brain regions in conflict resolution. In specific, the anterior cingulate cortex (ACC) (Leung et al., 2000; Peterson et al., 2002; Potenza et al., 2003; Langenecker et al., 2004), the bilateral dorsolateral prefrontal cortex (DLPFC) (Leung et al., 2000; Peterson et al., 2002; Potenza et al., 2003; Langenecker et al., 2004), the bilateral medial prefrontal cortex (mPFC) (Leung et al., 2000; Potenza et al., 2003; Langenecker et al., 2004), and the bilateral temporo-parietal junction (TPJ) (Peterson et al., 2002; Langenecker et al., 2004) have all shown increased activity during tasks which require cognitive conflict resolution.

Of interest, these regions are also associated with various meditative practices. Experienced meditators, for instance, show increased activity in the DLPFC, ACC, and mPFC during mindfulness (Hölzel et al., 2007; Baron Short et al., 2010). For those new to mindfulness, 3 days of training reportedly boosts the resting state functional connectivity between the DLPFC and frontoparietal control network regions (Taren et al., 2017). There is evidence that activation patterns during mindfulness change with mindfulness experience. Brefczynski-Lewis et al. (2007) showed that meditators with ∼19,000 h of experience had increased activity in the DLPFC and ACC during focused-attention meditation compared to novices. However, those with ∼40,000 h had reduced activation in these areas. Structural changes in the brain due to mindfulness have also been observed. Increase in white-matter density in the ACC in novices was reported after 6 weeks of mindfulness training (Tang et al., 2010). Greater gray matter density in the TPJ in novices was reported after attending an 8-week mindfulness program (Hölzel et al., 2011). While both neural changes from mindfulness and neural activity during conflict monitoring have been studied individually, the connection between neural activity in conflict resolution and mindfulness practices is still in early stages.

Functional magnetic resonance imaging (fMRI) and electroencephalography (EEG) are frequently used in mindfulness research, however, functional near-infrared spectroscopy (fNIRS) offers some advantages. Notably, fNIRS permits participants to be seated—a common position for meditation—whereas fMRI does not. Furthermore, fNIRS doesn’t produce the loud, disruptive noises associated with fMRI, which can be particularly distracting for novice meditators. Given these benefits, fNIRS was chosen as the imaging modality.

The current study was designed to investigate the long-term effects of mindfulness experience and the acute effects of a single mindfulness session on conflict resolution in experienced meditators and novices. It was hypothesized that experienced meditators would show better conflict resolution abilities compared to novices, and that experienced meditators would show less age-related decline in cognitive conflict resolution speed. In addition, it was expected that both experienced meditators and novices in the mindfulness condition would show greater improvements in conflict resolution skills compared to those in the control condition. Based on previous reports of mindfulness training increasing the activation of top-down attention related areas during conflict resolution (Allen et al., 2012), it was hypothesized that experienced meditators would show more activity in the dorsolateral prefrontal cortices and the temporoparietal junctions bilaterally during conflict resolution at baseline. It was also hypothesized that a 20-min mindfulness session would affect neural activity differentially across experienced meditators and novices, with experienced meditators in the mindfulness condition showing lesser activation in these regions during conflict resolution after intervention, and novices showing greater activation.

## 2 Materials and methods

### 2.1 Participants

Two participant groups were enrolled in the study: experienced meditators, with over 3 years of continuous mindfulness practice (at least 1 h weekly over the past 6 months), and novices, who had no regular mindfulness practice. Experienced meditators were recruited from various local meditation groups and Mid-City Zen Temple, and novices were recruited from the community. Groups were matched for age, gender, household income and education. For experienced meditators, having at least 1 h weekly meditation practice over the past 6 months and overall 3 years of meditation experience, was the only inclusion criteria. For novices, never having meditated regularly was the only inclusion criteria. For both groups left-handed participants were excluded. A total of 41 participants were recruited into the study, but 6 were excluded due to failures in following instructions in the Stroop task. A paired *t*-test revealed no statistical differences in age across both groups [Experienced Meditators: *M* = 38.61, SD = 11.35, Novices: *M* = 36.12 (15.03)]. Chi-square tests revealed no significant differences in the distributions of household income (HHI), education or gender levels across both groups (Table 1).

**TABLE 1:** Demographic data.

	Experienced meditators (*N* = 18)	Novices (*N* = 17)	Statistical tests
Age M (SD)	38.61 (11.35)	36.12 (15.03)	*U* = 198, *p* = 0.141
Gender (% female)	50%	53%	χ^2^ = 0, *p* = 1
Household Income (HHI) (Mode)	$100,000–150,000	$100,000–150,000	χ^2^ (8,40) = 7.177, *p* = 0.785
Education (Mode)	Master’s	Bachelor’s	χ^2^ (4, 40) = 7.177, *p* = 0.417

### 2.2 Procedure

**FIGURE 1:**
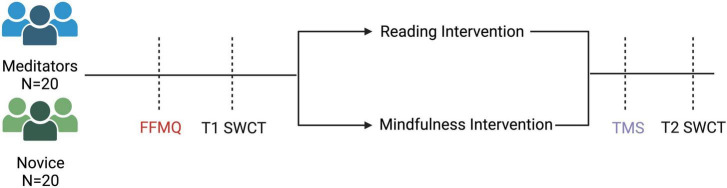
Experimental Procedure. Experienced meditators and novices completed the Five Facets of Mindfulness Questionnaire, then the Stroop task at T1. They were then assigned to a mindfulness intervention or a reading intervention. After the intervention, they completed the Toronto Mindfulness Scale, and then finally the Stroop task at T2.

The current study was conducted at the Tulane Brain Institute Human Research and Data Analysis Core, and was ethical approval was given by the Tulane Institutional Review Board (2020-1854-TUHSC). On arrival, participants provided demographic information, and completed the five facets of mindfulness questionnaire (FFMQ), which measured daily mindfulness. They were then fitted with an optode cap connected to a near-infrared spectroscopy (fNIRS) imager (NIRScout, NIRx Medical Technologies, LLC). The fNIRS montage comprised 24 Sources and 28 Detectors with 8 short-separation channels, covering frontal and temporal areas extensively (Figure 1). NIRStar acquisition software (NIRx Medical Technologies, LLC, New York, NY, USA) was used to record fNIRS data and assess the signal-to-noise ratio.

**FIGURE 2:**
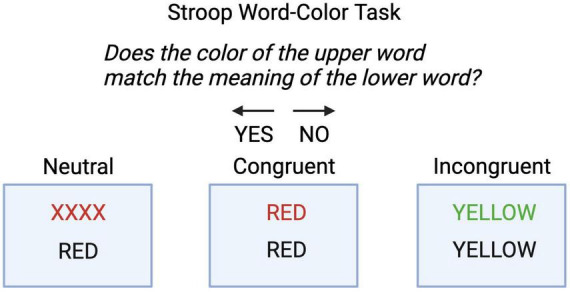
Stroop Word-Color Task. Participants were instructed to answer “Does the color of the upper word match the meaning of the lower word? They pressed the left arrow for “yes”, and right arrow for “no”. There were three conditions, neutral, congruent and incongruent.

Post calibration, participants completed the Stroop Word-Color Task (SWCT) (see Figures 1, 2), to assess baseline conflict monitoring (T1). The task consisted of 45 trials (15 per condition) with a 6-s inter-trial interval. SWCT used a block-design with congruent, neutral, and incongruent trials. A block-design was used to increase the power of detection of neural activation during each condition with fNIRS (Schaeffer et al., 2014). Reaction time, incongruency effect (reaction time difference between incongruent and neutral trials), accuracy during incongruent trials, and the correlation of these variables with age were the main behavioral outcome measures. Next, they were pseudo-randomly assigned to either a mindfulness condition or a reading condition.

In the mindfulness condition, the participants completed a breath count task, in which they counted their breathing cycles from 1 to 9, using the left arrow key for counting and the right arrow key to reset the count after the ninth breath. They pressed the spacebar if they lost count, which reset the counter, and started counting again using the left arrow. This intervention was reported to induce mindfulness (Levinson et al., 2014). In the control condition, participants read an auditory system-related text, pressing the right arrow key to navigate to the subsequent page (Hearing Link Services, 2011).

After the breath count or reading task, participants completed the Toronto Mindfulness Scale questionnaire (Lau et al., 2006) which was used as a manipulation check, and then completed the SWCT for the second time (T2) to assess the acute effects of the mindfulness intervention on cognitive conflict resolution. The second SWCT also consisted of 30 trials (10 per condition) with a 6-s inter-trial interval.

### 2.3 Statistical analysis

*T*-tests were performed to determine group differences between experienced meditators and novices based on their FFMQ scores, Stroop reaction time, incongruency effect, and accuracy in incongruent trials. Linear correlation between age and reaction time, incongruency effect and accuracy in incongruent trials were analyzed for experienced meditators and novices separately. The relationship between Stroop performance and age across experienced meditators and novices was further analyzed with three ANCOVAs for incongruency effect, accuracy in incongruent trials and reaction time, with age as a covariate.

For the manipulation check, experienced meditators and novices who completed the reading and breath count intervention were compared separately using a paired *t*-test, given their differing long-term mindfulness experiences. Breath count task performance was analyzed by comparing experienced meditators and novices on the number of correct breathing cycles, which were defined as eight left-button presses followed by a right button press, incorrect breathing cycles, and% of correct cycles with *t*-tests. Three two-way ANOVAs were performed to test Group and Time effects on Stroop incongruence, overall reaction time, and accuracy incongruent trials, using custom Python code, to determine the effect of the breath count intervention on cognitive conflict resolution performance.

Raw functional near-infrared spectroscopy (fNIRS) data was converted to optical density and then to chromophore concentration using nirs-toolbox functions (Santosa et al., 2018). fNIRS data was analyzed for both deoxy- and oxy-hemoglobin since oxy-hemoglobin concentration is expected to increase and deoxy-hemoglobin concentration is expected to decrease when a brain region is activated (Huppert et al., 2006). For the Stroop data, a general linear model (GLM) with auto-regressive iterative least squares (AR-IRLS) algorithm was used to calculate the beta-weights per Stroop condition per subject, per signal type (oxy- and deoxy-hemoglobin) (Barker et al., 2013; Jacques, 2013) and a mixed effects model was used to generate group averages for each variable combination (Expertise × Condition × Time × Trial). Breath count task fNIRS data was divided into three segments of equal length, beginning, middle and end for each subject. An AR-IRLS GLM was used to calculate beta weights per segment, per subject and a mixed effects model was used to generate group averages for experts and novices in the breath count conditions. For each analysis, outliers were removed using a built-in nirs-toolbox outlier removal function.

**FIGURE 3:**
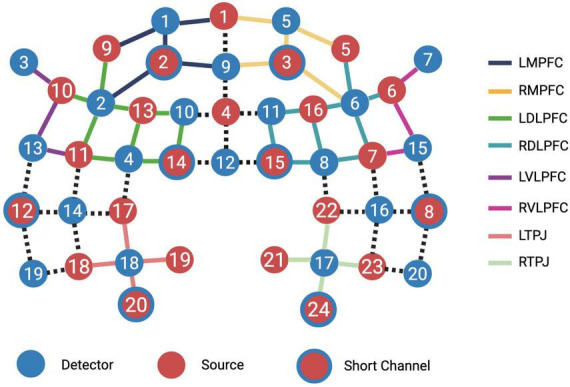
Region of interest (ROI) groupings. Individual channels were grouped into ROIs representing left and right medial prefrontal cortices, left and right dorsolateral prefrontal cortices, left and right ventrolateral prefrontal cortices and left and right temporoparietal junctions.

Channels were grouped into regions of interest (ROIs) (Figure 3) determined *a priori* and contrasts of interest were examined by *t*-test, and *p*-values for all fNIRS comparisons were corrected for multiple comparisons using the Benjamini-Hochberg procedure.

Linear correlation between RDLPFC, and LDLPFC activation and baseline Stroop incongruency (reaction time in incongruent trials–neutral trials) was analyzed as an exploratory analysis. The *p*-values for the correlation analysis were corrected for false discovery rate using Bonferroni’s adjustment.

## 3 Results

### 3.1 Behavioral results: effects of long-term mindfulness practice

#### 3.1.1 Five facets of mindfulness questionnaire

Experienced meditators demonstrated higher mindfulness levels on the Five-Facets of Mindfulness Questionnaire (FFMQ) than novices t(38) = 2.61, *p* = 0.013 (see Table 2).

**TABLE 2:** Baseline Stroop performance.

	Experienced meditators		Novices		Significance between groups
	M	SE	M	SE	
**FFMQ**	**143.33**	**2.98**	**130.24**	**4.14**	***p* = 0.016**
Stroop incongruency (sec)^a^	0.31	0.04	0.31	0.05	*p* = 0.96
Stroop reaction time (sec)	1.27	0.05	1.33	0.06	*p* = 0.50
Accuracy (%) in incongruent trials	90.74	2.23	85.49	3.19	*p* = 0.19

*^a^*Stroop Incongruency was calculated as reaction time for incongruent trials–neutral trials. Bold indicates significance at *p* < 0.05.

#### 3.1.2 Stroop task

There were no significant differences in Stroop reaction time, incongruency effect, or accuracy at baseline, suggesting that the participants’ prior mindfulness experience did not significantly influence conflict monitoring ability as assessed by the SWCT (see Table 2).

#### 3.1.3 Correlation of age and Stroop measures

**FIGURE 4:**
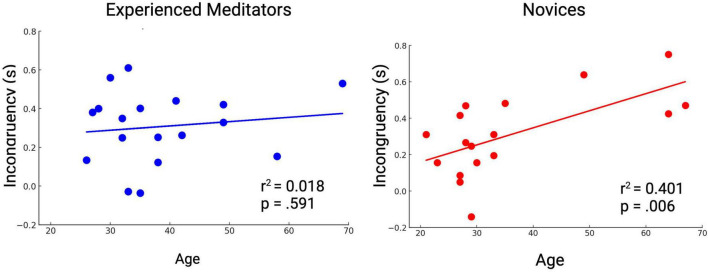
Novices show age-related decline in Stroop performance, while experienced meditators do not.

Novices demonstrated age-related decline in cognitive conflict resolution performance, as evidenced by an increased Stroop incongruency effect in older participants [r(15) = 0.633, *p* < 0.01]. This correlation wasn’t observed in experienced meditators [r(16) = 0.136, *p* = 0.591] (Figure 4). Both groups showed a correlation between reaction time and age, where older participants were slower during the Stroop task overall. [Novices r(15) = 0.514, *p* < 0.05, Experts r(16) = 0.591, *p* < 0.05].

ANCOVAs for the incongruency effect, reaction time and accuracy with Expertise as an independent variable and age as a covariate indicated a significant effect of age as a covariate for incongruency effect [*F*_(1,31)_ = 9.39, *p* = 0.0045], reaction time [*F*_(1,31)_ = 6.99, *p* = 0.013], and accuracy in incongruent trials [*F*_(1,31)_ = 4.35, *p* = 0.045]. Age x Expertise interaction wasn’t significant for any of the variables.

### 3.2 Behavioral results: manipulation check

Experienced meditators in the breath count condition scored higher on the Toronto Mindfulness Scale (TMS) curiosity subscale (*M* = 17.7, SE = 1.61) compared to those in the reading condition (*M* = 12.38, SE = 1.74), t(18) = 2.25, *p* = 0.04. This indicated that the breath count task induced a mindful curiosity state compared to the reading task. No significant differences were observed in the TMS openness subscale between experienced meditators assigned to either the reading or breath count interventions. However, experienced meditators across both groups scored higher on the openness subscale than novices (Experienced Meditators, *M* = 17.44, SE = 1.10, Novices, *M* = 12.53, SE = 1.19), indicating an effect of long-term mindfulness experience on openness, t(38) = 3.03, *p* < 0.01.

### 3.3 Behavioral results: effects of acute mindfulness practice

#### 3.3.1 Breath count task performance

Experienced meditators made less errors [t(17) = −2.27, *p* < 0.037] and had a higher percentage of correct trials [t(17) = 2.19, *p* < 0.044] in the breath count task compared to novices, indicating that they were better able to attend to their breath than novices.

#### 3.3.2 Change in Stroop performance T1 to T2

Across all participants, there was a significant main effect of Time on accuracy during incongruent trials *F*_(1,62)_ = 8.603, *p* < 0.01 and reaction time, *F*_(1,62)_ = 7.893, *p* < 0.01, thus, all participants improved their accuracy and reduced their reaction time from T1 to T2. There was no main effect of Group or Time × Group interaction in accuracy, reaction time, or incongruency (Table 3).

**TABLE 3:** T1 and T2 reaction time, incongruency and accuracy for all groups.

	Experienced meditator–Breath count		Experienced meditator–Reading control		Novices–Breath count		Novices–Reading control	
	T1	T2	T1	T2	T1	T2	T1	T2
Reaction time (sec)	1.30 (0.06)	1.08 (0.07)	1.24 (0.08)	1.09 (0.09)	1.40 (0.06)	1.25 (0.09)	1.26 (0.10)	1.13 (0.10)
Stroop incongruency^a^ (sec)	0.33 (0.06)	0.18 (0.07)	0.28 (0.07)	0.16 (0.03)	0.36 (0.09)	0.37 (0.08)	0.26 (0.05)	0.19 (0.04)
Accuracy in incongruent trials (%)	88.67 (3.45)	95.33 (2.44)	93.33 (2.52)	99.17 (0.83)	84.44 (3.69)	91.11 (4.01)	86.67 (5.63)	95.83 (2.50)

Mean and SE.

*^a^*Stroop Incongruency was calculated as reaction time for incongruent trials–neutral trials.

### 3.4 fNIRS results: manipulation check

During pre-processing, four participants were excluded from neural data analysis due to excessive noise in the signal.

The incongruent–neutral trial contrast across all participants indicated a decrease in deoxy-hemoglobin concentration in the left dorsolateral prefrontal cortex (LDLPFC) [t(162) = −4.79, *q* < 0.001], RDLPFC [t(162) = −4.62, *q* < 0.001], right medial prefrontal cortex (RMPFC) [t(162) = −5.02, *q* < 0.001], LMPFC [t(162) = −2.78, *q* < 0.05], left temporoparietal junction (LTPJ) [t(162) = −2.65, *q* < 0.05] and right ventrolateral prefrontal cortex (RVLPFC), [t(162) = −2.49, *q* < 0.05]. LVLPFC [t(162) = −4.21, *q* < 0.001] and RVLPFC [t(162) = −2.49, *q* < 0.05] showed a decrease in the oxy-hemoglobin signal in the same comparison. These results indicate a pattern of more DLPFC and MPFC activation during incongruent trials in comparison to neutral trials, as expected (see Table 4A).

**TABLE 4:** Incongruency effect at baseline (T1).

Region	Signal type	Beta coefficient	SE	T-statistic	*q*
**(A) All participants, incongruent > Neutral trials**					
L TPJ	HbR	−9.53	3.59	−2.65	<0.01
L MPFC	HbR	−7.23	2.60	−2.78	<0.01
R MPFC	HbR	−12.12	2.41	−5.02	<0.001
R VLPFC	HbR	−13.16	4.76	−2.76	<0.01
R DLPFC	HbR	−9.10	1.97	−4.62	<0.001
L DLPFC	HbR	−9.61	2.00	−4.79	<0.001
R VLPFC	HbO	−25.57	10.26	−2.49	<0.05
L VLPFC	HbO	−42.00	9.97	−4.21	<0.001
**(B) Incongruency effect at baseline, experienced meditators > Novices**					
R DLPFC	HbR	−5.69	1.97	−2.89	<0.05
L DLPFC	HbR	−8.60	2.00	−4.29	<0.001
R VLPFC	HbO	25.58	10.26	2.49	<0.05
R DLPFC	HbO	14.98	4.41	3.40	<0.01
L DLPFC	HbO	17.90	4.26	4.20	<0.01

### 3.5 fNIRS results: effects of long-term mindfulness practice

#### 3.5.1 T1 incongruency effect, experts vs. novices

In comparison with novices, experienced meditators showed a greater decrease in deoxy-hemoglobin concentration in the LDLPFC [t(162) = −4.29, *q* < 0.001] and RDLPFC [t(162) = −2.89, *q* < 0.05] for the neural incongruency effect (neural activity during incongruent trials–neural activity during neutral trials). They also showed a greater increase in oxy-hemoglobin concentration in the RVLPFC [t(162) = 2.49, *q* < 0.05], RDLPFC [t(162) = 3.39, *q* < 0.01] and LDLPFC [t(162) = 4.20, *q* < 0.001]. These results indicate more activation of LDLPFC, RDLPFC, and RVLPFC during cognitive conflict resolution among experienced meditators in comparison with novices (see Table 4B).

#### 3.5.2 T1, correlation of fNIRS and Stroop data

Correlation of RDLPFC and LDLPFC activation in incongruent trials with the incongruency effect at baseline was analyzed for experts and novices separately. Even though none of the effects survived correction for multiple comparisons, it is noteworthy that for experts, the decrease in deoxy-hemoglobin in the RDLPFC was positively correlated with incongruency, indicating greater RDLPFC activation was associated with decreased Stroop incongruency effect [r(16) = 0.624, *p* = 0.007]. For Novices, same was true for the LDLPFC [r(13) = 0.647, *p* = 0.014].

### 3.6 fNIRS results: effects of acute mindfulness practice

#### 3.6.1 Neural activity during the breath count task, within experts

Experienced meditators showed increases from baseline in oxy-hemoglobin concentration in the LVLPFC and the RVLPFC during the middle segment of the breath count task (LVLPFC [Table 5A, t(162) = 3.12, *q* < 0.05], RVLPFC [Table 5A, t(162) = 4.06, *q* < 0.01] Experienced meditators also showed a decrease from baseline in the oxyhemoglobin concentration in the LTPJ [mid-segment: Table 5A, t(162) = −3.06, *q* < 0.05, end-segment: Table 5B, t(162) = −3.01, *q* < 0.05].

**TABLE 5:** Oxy- and Deoxy-hemoglobin beta values during breath count task for experienced meditators and novices.

Region	Signal type	Beta coefficient	SE	T-statistic	*q*
**(A) Experienced meditators, regions significantly different from baseline, middle portion of breath count task**					
L VLPFC	HbR	−14.12	4.87	−2.90	<0.05
R DLPFC	HbR	5.15	1.88	2.73	<0.05
L TPJ	HbO	−20.73	6.78	−3.06	<0.05
R TPJ	HbO	−34.15	8.23	−4.15	<0.01
**R VLPFC**	**HbO**	**30.78**	**7.59**	**4.06**	**<0.01**
L VLPFC	HbO	27.88	8.93	3.12	<0.05
**(B) Experienced meditators, regions significantly different from baseline, end portion of breath count task**					
**R VLPFC**	**HbR**	−**12.26**	**4.37**	−**2.81**	**<0.05**
L VLPFC	HbR	−17.20	5.32	−3.23	<0.05
L DLPFC	HbR	−6.72	1.81	−3.71	<0.01
L TPJ	HbO	−22.04	7.33	−3.01	<0.05
R TPJ	HbO	−32.57	8.52	−3.82	<0.05
**(C) Novices, regions significantly different from baseline, middle portion of breath count task**					
**R MPFC**	**HbR**	**8.18**	**2.72**	**3.00**	**<0.05**
R MPFC	HbO	−19.63	7.87	−2.49	<0.05
**R VLPFC**	**HbO**	−**43.96**	**16.94**	−**2.60**	**<0.05**
**(D) Novices, regions significantly different from baseline, end portion of breath count task**					
RMPFC	HbR	12.13	3.19	3.81	<0.01
R TPJ	HbO	−32.41	10.99	−2.95	<0.05
R MPFC	HbO	−36.34	8.86	−4.10	<0.01
**R VLPFC**	**HbO**	−**47.98**	**18.73**	−**2.56**	**<0.05**
**R DLPFC**	**HbO**	−**22.11**	**8.50**	−**2.60**	**<0.05**

Bold indicates significant difference between groups.

Experienced meditators showed significant decreases in deoxy-hemoglobin concentrations in the LDLPFC [mid-segment: Table 5A, t(162) = −3.71, *q* < 0.01], RVLPFC [mid-segment: Table 5A, t(162) = −2.81, *q* < 0.01] and LVLPFC [mid-segment: Table 5A, t(162) = −2.90, *q* < 0.01, end-segment: Table 5B, t(162) = −3.23, *q* < 0.01] during the breath count task. These patterns of activation are consistent with increased frontal activity during the breath count task for experienced meditators.

#### 3.6.2 Neural activity during the breath count task, within novices

Novices in the breath count task showed decreases in oxy-hemoglobin concentration in the RDLPFC [end-segment: Table 5D, t(162) = −2.60, *q* < 0.05], RMPFC [mid-segment: Table 5C, t(162) = −2.50, *q* < 0.05, end-segment: Table 5D, t(162) = −4.1, *q* < 0.01], RVLPFC [mid-segment: Table 5C, t(162) = −2.60, *q* < 0.05, end-segment: Table 5D, t(162) = 2.61, *q* < 0.05] and RTPJ [end-segment: Table 5D, t(162) = −2.95, *q* < 0.05]. Deoxy-hemoglobin concentrations significantly increased in the RMPFC [mid-segment: Table 5C, t(162) = 3.00, *q* < 0.05, end-segment: Table 5D, t(162) = 3.81, *q* < 0.01]. These patterns of activation are consistent with no change in activation in frontal regions during the breath count task for novices.

#### 3.6.3 Neural activity during the breath count task, experts vs. novices

Experienced meditators showed higher oxy-hemoglobin concentrations than novices in the RDLPFC [end-segment: t(162) = 2.75, *q* < 0.05] and RVLPFC [beginning-segment: t(162) = 2.70, *q* < 0.01, mid-segment: t(162) = 4.03, *q* < 0.01, end-segment: t(162) = 3.31, *q* < 0.05]. Experienced meditators also showed less deoxy-hemoglobin concentrations than novices in the RMPFC in the end-portion of the breath count task. [end-segment: t(162) = −2.51, *q* < 0.05]. These results indicate that experienced meditators showed more frontal activation during the breath count task in comparison to novices, especially in the middle and end segment.

#### 3.6.4 T1 to T2 change in incongruency effect, novices in the breath count condition

Novices in the breath count condition showed increases in oxy-hemoglobin in the RDLPFC [Table 6A, t(162) = 4.88, *q* < 0.001], LDLPFC [Table 6A, t(162) = 4.41, *q* < 0.001], RVLPFC [Table 6A, t(162) = 2.61, *q* < 0.05], LVLPFC [Table 6A, t(162) = 2.93, *q* < 0.05], RMPFC [Table 6A, t(162) = 4.59, *q* < 0.001], and LMPFC [Table 6A, t(162) = 2.67, *q* < 0.05] and RTPJ [Table 6A, t(162) = 4.52, *q* < 0.001], during conflict resolution at T2 in comparison to conflict resolution at T1. Novices also showed decreases in deoxy-hemoglobin in the LVLPFC [Table 6A, t(162) = −2.91, *q* < 0.05] and LDLPFC [Table 6A, t(162) = −2.98, *q* < 0.05].

**TABLE 6:** Incongruency effect T2–T1, all four groups.

Region	Signal type	Beta coefficient	SE	T-statistic	*q*
**(A) Novices breath count, incongruency effect, T2 > T1**					
L VLPFC	HbR	−14.77	5.08	2.91−	<0.05
L DLPFC	HbR	−3.90	1.31	−2.98	<0.05
R TPJ	HbO	25.40	5.62	4.52	<0.001
L MPFC	HbO	11.04	4.14	2.67	<0.05
R MPFC	HbO	18.43	4.02	4.59	<0.001
R VLPFC	HbO	20.41	7.81	2.61	<0.05
L VLPFC	HbO	24.36	8.32	2.93	<0.05
R DLPFC	HbO	15.81	3.23	4.89	<0.001
L DLPFC	HbO	11.91	2.70	4.41	<0.001
**(B) Experienced meditators breath count, incongruency effect, T2 > T1**					
L TPJ	HbR	7.67	2.33	3.30	<0.01
L VLPFC	HbR	−7.92	3.09	−2.57	<0.05
R DLPFC	HbR	2.76	1.12	2.47	<0.05
L DLPFC	HbR	3.23	1.16	2.78	<0.05
L TPJ	HbO	−12.58	4.42	−2.85	<0.05
R MPFC	HbO	−9.67	3.07	−3.15	<0.05
R VLPFC	HbO	17.23	5.40	3.19	<0.05
L VLPFC	HbO	28.20	6.09	4.63	<0.001
L DLPFC	HbO	7.58	2.42	3.13	<0.05
**(C) Novices reading control, incongruency effect, T2 > T1**					
L DLPFC	HbR	−9.00	3.19	−2.83	<0.05
R VLPFC	HbO	30.51	8.80	3.47	<0.01
L VLPFC	HbO	22.61	6.72	3.36	<0.01
R DLPFC	HbO	10.57	3.04	3.48	<0.01
**(D) Experienced meditators reading control, incongruency effect, T2 > T1**					
R DLPFC	HbO	9.87	3.02	3.27	<0.05

#### 3.6.5 T1 to T2 change in incongruency effect, novices in the reading condition

Novices in the reading condition showed increases in oxy-hemoglobin concentration in the RDLPFC [Table 6C, t(162) = 3.48, *q* < 0.01], RVLPFC [Table 6C, t(162) = 3.47, *q* < 0.01], and LVLPFC [Table 6C, t(162) = 3.36, *q* < 0.01]. They also showed a decrease in the deoxy-hemoglobin concentration in the LVLPFC [Table 6C, t(162) = −2.83, *q* < 0.05].

#### 3.6.6 T1 to T2 change in incongruency effect, experienced meditators in the breath count condition

Experienced meditators in the breath count condition showed decreases in oxy-hemoglobin concentration in the LTPJ [Table 6B, t(162) = 4.88, *q* < 0.001] and the RMPFC [Table 6B, t(162) = −3.15, *q* < 0.05], and increases in oxy-hemoglobin concentration in the RVLPFC [Table 6B, t(162) = 3.19, *q* < 0.05], LVLPFC [Table 6B, t(162) = 4.63, *q* < 0.001],and LDLPFC [Table 6B, t(162) = 3.13, *q* < 0.05]. They also showed increases in deoxy-hemoglobin concentrations in the LTPJ [Table 6B, t(162) = 3.30, *q* < 0.01], RDLPFC [Table 6B, t(162) = 2.47, *q* < 0.05], and LDLFPC [Table 6B, t(162) = 2.78, *q* < 0.05].

#### 3.6.7 T1 to T2 change in incongruency effect, experienced meditators in the reading condition

Experienced meditators in the reading condition showed an increase in oxy-hemoglobin concentration in the RDLPFC [Table 6D, t(162) = 3.27, *q* < 0.05] during conflict resolution in T2 in comparison to T1.

#### 3.6.8 T1 to T2 change in incongruency effect, experienced meditators vs. novices in the breath count condition

At T1, experienced meditators assigned to the breath count task demonstrated more activation in the RDLPFC and LDLPFC compared to novices in the breath count task [RDLPFC: t(162) = 3.88, *q* < 0.01, LDLPFC: t(162) = 3.85, *q* < 0.01]. At T2 experienced meditators still demonstrated more activation in the LDLPFC [t(162) = 2.69, *q* < 0.03], but the difference was reversed in the RDLPFC where novices showed more activation during conflict resolution at T2 [t(162) = −3.40, *q* < 0.01].

**FIGURE 5:**
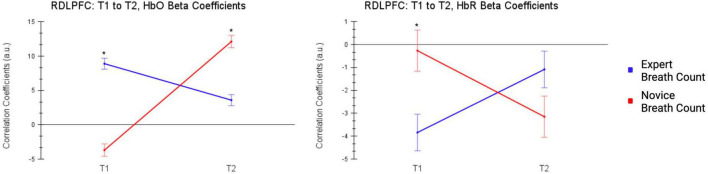
T1 to T2, Changes in activation of RDLPFC during conflict resolution (incongruent - neutral trials) in experienced meditators and novices in the breath count groups from T1 to T2. A brief mindfulness session differentially impacts RDLPFC activation during conflict resolution in novices and experienced meditators.

Compared to novices in the breath count condition, Experienced meditators in the breath count condition showed greater decreases in deoxy-hemoglobin during conflict resolution at T1 inRDLPFC: t(162) = −2.77, *q* < 0.05, and LDLPFC: t(162) = −4.98, *q* < 0.001). There were no significant differences in the deoxy-hemoglobin signal between the two groups at T2. This pattern indicates that a brief mindfulness intervention increased neural activation during conflict resolution in novices, making them more similar to experienced meditators (Figure 5).

## 4 Discussion

The current study examined effects of long-term mindfulness experience and a single mindfulness session on regional cortical activity and cognitive conflict resolution in experienced meditators and novices.

Among novices, cognitive conflict resolution speed was related to age, whereas this relationship was not evident among experienced meditators. This indicates that long-term mindfulness practice may protect against age-related cognitive decline, which is consistent with previous reports (Pagnoni and Cekic, 2007; Prakash et al., 2012; Sperduti et al., 2016).

It was hypothesized that at baseline (T1), experienced meditators with 3 + years of meditation experience, and 6-months of continuous practice, would show more neural activity in the bilateral dorsolateral prefrontal cortices (DLPFC) and temporoparietal junctions (TPJ) during conflict resolution compared to novices. In addition, it was hypothesized that experienced meditators would show better conflict resolution performance (less Stroop incongruency effect) compared to novices. The findings of the current study support this hypothesis with regard to neural activation for the DLPFC bilaterally, but no group differences were found in the TPJ. Contrary to our hypotheses, there were no significant differences between experienced meditators and novices in Stroop reaction time, incongruency effect, or accuracy on incongruent trials at T1. This finding is consistent with reports of null findings (Kozasa et al., 2012; Lykins et al., 2012), but not with reports of reduced Stroop incongruency effect in experienced meditators when compared to novices (Chan and Woollacott, 2007; Moore and Malinowski, 2009; Fabio and Towey, 2018). Discrepant findings among studies of mindfulness practices may be related to differences in levels of experience or expertise. Fabio and Towey (2018) defined experienced meditators as those with at least 6 years of experience, and reported a significant difference between cognitive resolution. However, Lykins et al. (2012) defined expertise criteria with at least a 6 month continuous practice, and reported null findings. The current study defined experienced meditators as participants with 3 years of experience and 6 months of continuous practice, and found increased DLPFC activation during conflict monitoring, with no behavioral difference. These outcomes suggest that the benefits of mindfulness on conflict resolution may be more evident after at least 3 years of experience, and that changes in neural activity may precede these benefits. A systematic study of groups of meditators with different amounts of experience may be useful to determine a timeline for the benefits of mindfulness experience on cognitive conflict resolution.

It is important to note that some previous reports indicate less neural activation during conflict resolution in experts with 3 + years of meditation experience (Kozasa et al., 2012, 2018). In particular, these reports indicate decreased activation in the medial-prefrontal/ACC clusters during conflict resolution in experienced meditators in comparison to novices, measured via functional magnetic resonance imaging. This discrepancy with the current results might be due to the differences in expertise criteria in the current study. While Kozasa et al. (2012, 2018) included only participants with 3 + years of regular practice, the current study included participants with 3 + years of practice, but only required 6 months of regular, continuous practice. Neural activity in the left DLPFC during mindfulness has been shown to follow an inverse U shape, with experts with an average of 19,000 h showing more activation, but experts with an average of 48,000 h showing less activation in comparison to novices, during mindfulness (Brefczynski-Lewis et al., 2007). This same pattern might apply to activity during cognitive conflict resolution as a result of mindfulness training.

During the mindfulness intervention, experienced meditators demonstrated an increase in neural activation in ventrolateral and dorsolateral prefrontal regions, while novices demonstrated no change in activation. The increase demonstrated by experienced meditators is consistent with previous findings (Brefczynski-Lewis et al., 2007; Hölzel et al., 2007; Baron Short et al., 2010; Deepeshwar et al., 2015), and indicates increased engagement of the dorsal-attention network in experienced meditators during mindfulness. Novices likely weren’t able to engage in the mindfulness task in the same way as experienced meditators, therefore demonstrating no change in activation in the same prefrontal regions. This may indicate a shift toward more mind-wandering in comparison to focused-attention, therefore activation of default mode network (DMN) rather than frontoparietal network (FPN) during the mindfulness task (Hasenkamp et al., 2012). The current behavioral results demonstrating fewer errors in counting breaths for experienced mediators compared to novices also support this conclusion.

Novices in the mindfulness condition showed increased activation of the bilateral DLPFC, MPFC, and VLPFC during conflict resolution at T2 in comparison to T1. Experienced meditators in the mindfulness condition showed increased activation in the VLPFC bilaterally, and decreased activation in the RMPFC and RDLPFC. Oxy-hemoglobin and deoxy-hemoglobin concentrations both increased for the LDLPFC in experienced meditators. Oxy-hemoglobin signals are expected to increase and deoxy-hemoglobin signals are expected to decrease during neural activation. Therefore the change in LDLPFC activation was not interpreted, and was likely due to physiological noise. A direct comparison of experienced meditators and novices in the breath count task indicated that experienced meditators showed more activation in the RDLPFC at T1, but at T2, novices showed more activation in the RDLPFC compared to experienced meditators after the intervention (see Figure 5). These results demonstrate the differential impact of mindfulness on cognitive conflict resolution, and indicate that novices became more similar to experienced meditators in neural activity after one mindfulness intervention.

The finding that novices showed no activation in prefrontal regions during the mindfulness intervention, but increased activation during conflict resolution after the mindfulness intervention might seem to be in conflict. However, it is important to note that these two effects are on different timescales. The mindfulness task was analyzed in three segments, each ∼7 min, detecting average change over that period of time, whereas conflict resolution was the difference of incongruent and neutral trials, ∼90 s. It is conceivable that even though novices engaged prefrontal regions of the frontoparietal network during the mindfulness task, this effect wasn’t visible in the whole block, especially considering the sensitivity of fNIRS to changes in systemic physiology. The current study didn’t have the ability to identify moments of focused-awareness in comparison to mind-wandering, therefore analysis was confined to relatively large segmentation of the mindfulness task. Therefore, novices might have engaged areas related to the frontoparietal network (FPN), such as the DLPFC, during mindfulness, priming these areas to be more active during conflict resolution at T2. Experienced meditators were likely more effective in engaging these areas during mindfulness and therefore showed a significant increase in activation, even in the longer blocks. In addition, our findings that experienced mediators showed decreased activation in RDLPFC and RMPFC during conflict resolution after mindfulness demonstrate differential change in neural activation as a result of mindfulness depending on mindfulness experience. This decrease in activation might indicate increased efficiency in processing conflict resolution in experienced meditators (Kozasa et al., 2012).

The current study has certain limitations. Functional near-infrared spectroscopy (fNIRS), while suitable for a naturalistic setting for meditation, is limited to examination of cortical areas. In addition, fNIRS measures brain activity by emitting light onto the scalp’s surface and is sensitive to variations in the brain region’s cortical depth relative to the scalp, as well as by external factors such as the thickness of the skin, fat, skull, and cerebrospinal fluid. These factors weren’t controlled for, given the researchers didn’t have access to structural MRI scans from the participants. The current study also employed a single cognitive conflict resolution measure, SWCT, given extensive reports of studies using a version of this task. A single cognitive conflict resolution task was also chosen to minimize the duration of the experiment: the fNIRS probes were found to cause mild discomfort when worn for long durations. Future studies might benefit from using a variety of tasks with different levels of difficulty.

In summary, the current findings provide evidence that a single mindfulness session alters regional cortical neural activity differentially across experienced meditators and novices, and that long-term mindfulness experience might protect against age-related decline in cognitive conflict resolution.

## Data availability statement

The raw data supporting the conclusions of this article will be made available by the authors, without undue reservation.

## Ethics statement

The studies involving humans were approved by the Tulane Institutional Review Board. The studies were conducted in accordance with the local legislation and institutional requirements. The participants provided their written informed consent to participate in this study.

## Author contributions

DE: Conceptualization, Data curation, Formal Analysis, Methodology, Project administration, Visualization, Writing – original draft, Writing – review and editing. MV: Conceptualization, Methodology, Writing – review and editing. PC: Conceptualization, Funding acquisition, Methodology, Project administration, Resources, Supervision, Writing – review and editing.
